# Graphitic Carbon Nitride in Oral Health: Emerging Applications, Antimicrobial Potential, and Future Perspectives

**DOI:** 10.3390/ijms262411860

**Published:** 2025-12-09

**Authors:** Gertrud Alexandra Paltinean, Marioara Moldovan, Codruta Sarosi, Laura Silaghi-Dumitrescu, Stanca Cuc, Gabriel Furtos, Ioan Petean, Irina Camelia Chis

**Affiliations:** 1 Raluca Ripan Institute for Research in Chemistry, Babeș-Bolyai University, 30 Fantanele Street, 400294 Cluj-Napoca, Romania; gertrud.paltinean@ubbcluj.ro (G.A.P.); laura.silaghi@ubbcluj.ro (L.S.-D.); stanca.boboia@ubbcluj.ro (S.C.); gfurtos@yahoo.co.uk (G.F.); 2 Faculty of Chemistry and Chemical Engineering, Babes-Bolyai University, 11 Arany János Street, 400028 Cluj-Napoca, Romania; ioan.petean@ubbcluj.ro; 3 Department of Physiology, Iuliu Hatieganu University of Medicine and Pharmacy, 1-3 Clinicilor Street, 400006 Cluj-Napoca, Romania; ichis@umfcluj.ro

**Keywords:** graphitic carbon nitride, atomic force microscopy (AFM), scanning tunneling microscopy (STM), nanostructures, properties

## Abstract

This comprehensive review highlights the growing significance of graphitic carbon nitride (g-C_3_N_4_) as a multifunctional material with applications spanning diverse scientific and technological domains. Based on an extensive literature from electronic databases such as Web of Science, PubMed and Google Scholar, we provide an in-depth discussion of the fundamental structural configurations of g-C_3_N_4_, namely the triazine- and heptazine-based frameworks, which form the basis of its unique physicochemical and electronic properties. The two predominant synthesis strategies—thermal polymerization and solvothermal/hydrothermal routes—are examined in detail and illustrated through representative schematic models to elucidate their underlying mechanisms and resulting structural variations. Advanced surface characterization techniques, such as atomic force microscopy (AFM) and scanning tunneling microscopy (STM), are also discussed in the context of their application to materials, including Highly Oriented Pyrolytic Graphite (HOPG), graphene oxide, and carbon nitride. These analyses provide insights into nanoscale surface topography and structural attributes, with HOPG serving as a well-established reference material for comparison. The review also addresses the biological activities and potential applications of g-C_3_N_4_, particularly in the context of its photocatalytic, antimicrobial, and biocompatible properties. Despite substantial progress in other research fields, a notable gap remains in the exploration of g-C_3_N_4_ for oral and dental applications. This limitation is largely attributed to the scarcity of systematic studies and limited published data in this emerging area. Accordingly, this review identifies promising opportunities for future research aimed at harnessing the distinctive properties of g-C_3_N_4_ for innovative developments in oral healthcare and dental material science.

## 1. Introduction

This review began with a systematic search of electronic databases, including Web of Science, PubMed, and Google Scholar, using keywords such as “g-C_3_N_4_in dentistry” and “g-C_3_N_4_ in dental materials”. The inclusion criteria focused on studies published within the last 10 years, available in full text, and written in English. These parameters ensured that the methodology and results could be thoroughly evaluated. As for exclusion criteria, the review omitted articles available only as abstracts, as well as news items, letters, interviews, and publications written in languages other than English.

The selection process was conducted in two steps. First, titles and abstracts were evaluated, and only articles addressing the application of g-C_3_N_4_ in dental composite materials were retained. Subsequently, a full-text screening was performed, during which the selected articles underwent a comprehensive review to verify their adherence to the predefined eligibility criteria.

**Figure 1:**
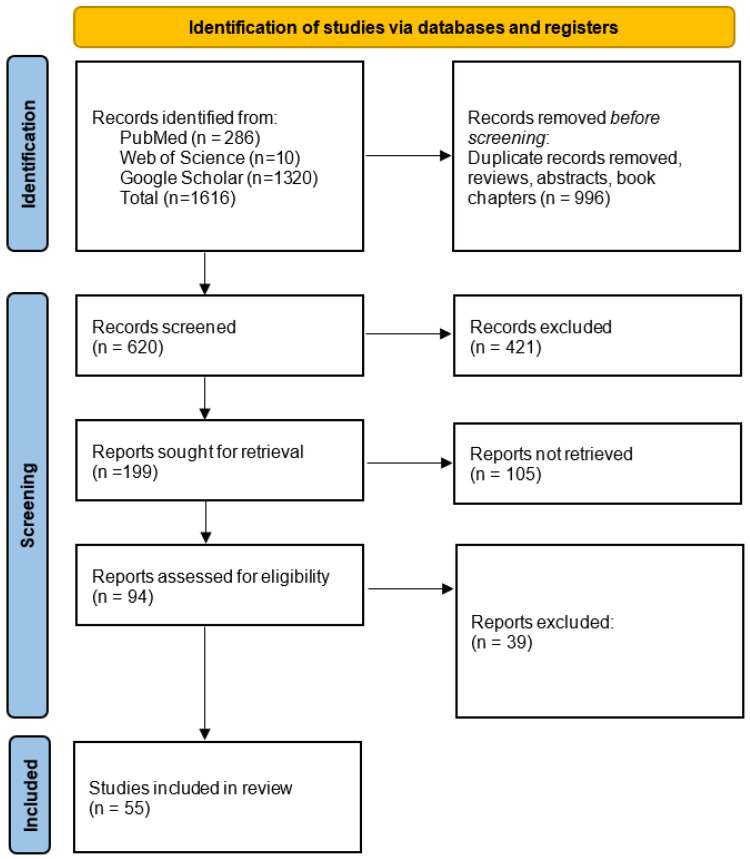
Prisma flow diagram.

Following selection, the review was structured around key aspects, including the synthesis, morphology, properties, and applications of g-C_3_N_4_in dental composite materials. The methodological quality of the included studies was assessed using standardized tools, such as the PRISMA framework (Figure 1).

The field of oral health is continually evolving, driven by dental practitioners refining their clinical expertise and by rapid advancements in materials science and biomedical technology. Oral health plays a vital role in maintaining overall well-being, necessitating the adoption of effective strategies for the prevention and management of oral diseases, as well as the promotion of healthy lifestyles through education and improved accessibility to dental care, particularly for underserved populations. Early prevention and timely treatment of oral pathologies are essential to achieving simpler, more effective interventions that benefit both patients and dental professionals engaged in restorative and therapeutic procedures.

**Figure 2:**
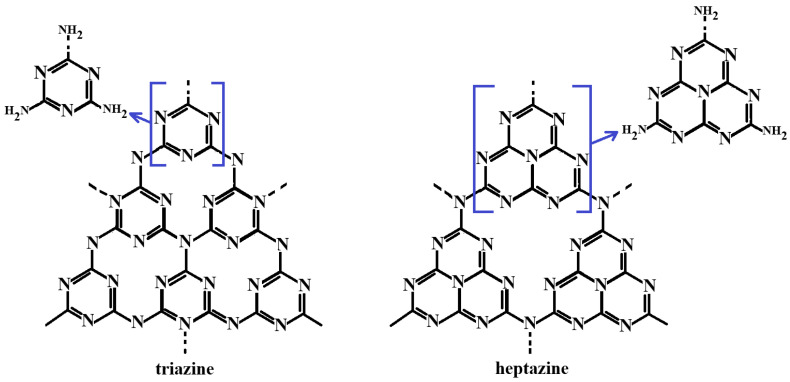
Structural units of g-C_3_N_4_ [8,9,10,11].

Within this dynamic context, graphitic carbon nitride (g-C_3_N_4_) has emerged as an innovative and promising material that has attracted increasing scientific attention in recent years. Characterized by low toxicity and excellent biocompatibility, g-C_3_N_4_ exhibits a high specific surface area, efficient charge transfer, strong photocatalytic activity, and remarkable thermal and chemical stability. Owing to its structural similarity to graphene, this material has found widespread applications across numerous scientific disciplines [1,2,3]. Theoretical studies attribute these unique properties to its atomic composition of carbon and nitrogen, arranged in triazine (C_3_N_3_) and heptazine (C_6_N_7_) units, as illustrated in Figure 2.

The s-triazine and tri-s-triazine rings forming the heptazine structure are organized into two-dimensional layers, interconnected by covalent bonds between sp^2^-hybridized carbon and nitrogen atoms [4,5,6,7]. This configuration yields an extended, sheet-like polymeric framework composed of infinite layers bound by weak van der Waals forces and π–π interactions. The presence of nitrogen atoms partially impedes electron delocalization across the layers, influencing the material’s electronic and optical behavior [4].

The resulting honeycomb-like atomic arrangement confers exceptional thermal stability, up to approximately 600 °C, along with high chemical resistance to environmental agents, acids, bases, and organic solvents [4,12].

**Figure 3:**
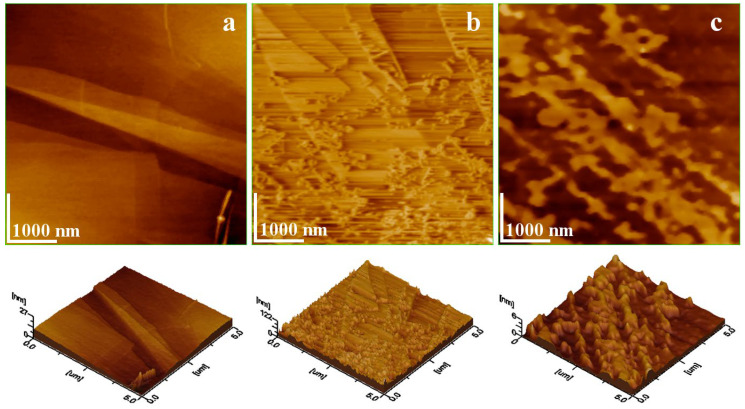
Atomic Force Microscopy (AFM) topographic images of (**a**) HOPG, (**b**) Graphene oxide sheets and (**c**) Graphite nitride. The three-dimensional profiles are given below each topographic image.

Theoretical modeling of graphite-based structures reveals that g-C_3_N_4_ derivatives originate from the hexagonal crystalline structure of pure graphite. Among these, Highly Oriented Pyrolytic Graphite (HOPG) represents the most ordered and structurally reliable form of graphite [13]. It is composed of perfectly aligned hexagonal carbon sheets with atomic-level smoothness and minimal surface roughness, exhibiting an average local height of approximately 27 nm (Figure 3a). Partial cleavage along interlayer planes results in single-atomic carbon layers, forming graphene while maintaining the hexagonal framework [14]. Subsequent oxidation increases surface roughness due to sheet corrugation (Figure 3b), raising the local height to around 122 nm. The edges of these ruffled layers contain reactive functional groups, enhancing their suitability for use as interfacial mediators in composite systems by facilitating bonding between polymer matrices and fillers.

In contrast, nitrogen incorporation into graphite layers produces a distinct topographical modification compared with oxidation. The intercalation of nitrogen atoms within the hexagonal carbon lattice causes localized ruptures of extended sheets and the formation of fine microstructural islands with significantly reduced height, around 6 nm (Figure 3c). This micro- and nano-scale structuring provides notable advantages:

- The ultrathin morphology enables effective dispersion in liquid media, allowing g-C_3_N_4_ sheets to interact with filler particles before polymerization, thereby promoting well-organized composite microstructures.
- The moderate reactivity of graphite nitride offers greater process control than graphene oxide, which is often excessively reactive.

**Figure 4:**
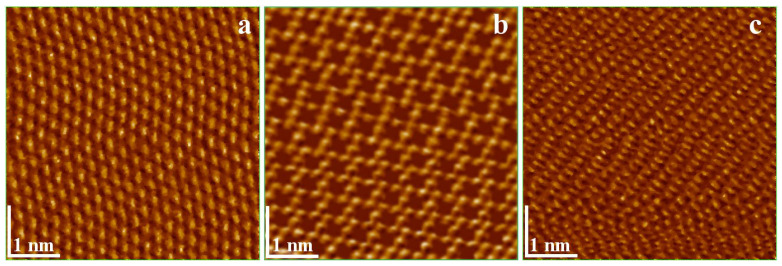
Scanning Tunneling Microscopy (STM) images taken at atomic resolution for: (**a**) HOPG, (**b**) Graphene oxide sheets and (**c**) Graphite nitride.

The distinctive properties of these graphite-derived compounds arise from their atomic arrangements within the crystalline lattice [15,16]. Scanning Tunneling Microscopy (STM) enables direct observation of these atomic structures (Figure 4). HOPG exhibits a hexagonal close-packed arrangement, with six carbon atoms positioned at the vertices of a regular hexagon and one atom intercalated between adjacent layers (Figure 4a). Cleavage of a single atomic layer produces graphene, which develops periodic voids upon oxidation, thereby enhancing its surface reactivity (Figure 4b). Conversely, nitrogen atom insertion into the HOPG lattice induces local distortions due to the slightly smaller atomic radius of nitrogen compared to carbon (Figure 4c).

This review provides an integrated overview of the fundamental aspects of graphitic carbon nitride, including its composition, structure, and major synthesis methodologies employing various precursors that influence its morphology and functionality. Furthermore, it explores the incorporation of g-C_3_N_4_ into composite materials with potential applications in oral and dental science. Several studies have investigated the incorporation of graphene into dental composites, tailoring these materials for specific dental applications. These approaches have demonstrated notable enhancements in mechanical properties, biocompatibility, and antimicrobial performance [17,18,19,20,21]. The insights presented herein highlight new opportunities for enhancing the efficiency, performance, and applicability of g-C_3_N_4_-based materials within the field of oral healthcare.

## 2. Synthesis of Graphitic Carbon Nitride

Although carbon is one of the most abundant elements on Earth, graphitic carbon nitride (g-C_3_N_4_) is not found naturally and must be synthesized artificially under controlled laboratory conditions. Among the various synthesis routes explored, thermal polymerization remains the most widely employed technique due to its simplicity, low cost, and versatility.

### 2.1. Thermal Polymerization

**Figure 5:**
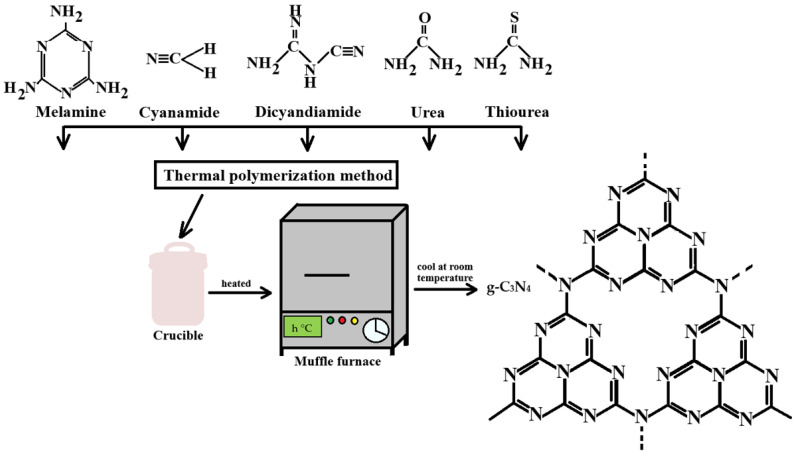
Schematic representation using nitrogen-rich precursors to produce g-C_3_N_4_ [22,23,25,26].

This method, extensively described in the literature, utilizes inexpensive nitrogen-rich precursors such as melamine, cyanamide, urea, or thiourea, as schematically represented in Figure 5. The synthesis process involves heating these precursors to temperatures typically ranging between 500 °C and 600 °C in an inert atmosphere, leading to the formation of the characteristic polymeric framework of g-C_3_N_4_ [22,23,24].

The type of precursor—used individually or in combination—has a significant influence on the structural and physicochemical properties of the resulting material, thereby affecting its performance and suitability for specific applications [4,22,23,24]. Table 1 provides representative examples of g-C_3_N_4_ synthesized from various precursors.

Huang et al. [27] synthesized g-C_3_N_4_ via thermal polymerization using melamine and urea. Their findings demonstrated that melamine tends to produce bulk g-C_3_N_4_ aggregates with minimal porosity, whereas urea favors the formation of irregular, layered architectures. This structural variation arises from high-temperature calcination, which facilitates urea polymerization and the generation of two-dimensional (2D) structures with enhanced textural porosity [27,28,30,33,34].

Molai and Rahimi-Moghadam [29] prepared g-C_3_N_4_ through the thermal polycondensation of melamine at temperatures ranging from 450 °C to 700 °C, employing heating rates of 2, 5, and 10 °C min^−1^. Their results revealed that g-C_3_N_4_ formation remains incomplete at 450 °C, while higher temperatures promote structural growth and network expansion. Optimal synthesis conditions were achieved at 700 °C with a heating rate of 2 °C min^−1^, yielding a material with a specific surface area of 38.38 m^2^ g^−1^ [29].

In another study, Huang et al. [31] employed dicyandiamide as a precursor and subjected it to pyrolysis at 550 °C, with heating rates of 5, 10, 15, and 20 °C min^−1^ for durations of 1, 2, and 4 h. The resulting materials exhibited a transformation from dense to porous structures as the heating rate increased. The optimal conditions—15 °C min^−1^ for 2 h—produced a porous g-C_3_N_4_ characterized by a high specific surface area, stable chemical bonding, and abundant amino functional groups [31].

Alwin et al. [32] confirmed the formation of poly-heptazine chains within g-C_3_N_4_ structures using X-ray Photoelectron Spectroscopy (XPS) and X-ray Diffraction (XRD), while also noting that higher calcination temperatures enhance both the degree of condensation and the structural order of the material [32]. Similarly, Xia et al. [30] investigated a range of precursors—including urea, guanidine hydrochloride, dicyandiamide, melamine, and thiourea—and reported pronounced morphological differences among the resulting products: melamine yielded aggregated particles, dicyandiamide produced sheet-like or granular morphologies, whereas urea generated laminar architectures with larger specific surface areas [30,35].

Thiourea, in particular, exhibited a strong dependence on the polymerization temperature. Hong et al. [36] investigated synthesis temperatures of 540 °C, 560 °C, and 580 °C at a constant heating rate of 2.3 °C min^−1^, observing that only the sample prepared at 580 °C formed well-defined 2D structures [36]. Furthermore, comparative studies indicate that precursors such as dicyandiamide, cyanamide, and melamine tend to produce mechanically hard g-C_3_N_4_ frameworks, while urea and thiourea yield softer and more flexible materials [4].

The mechanistic pathway of g-C_3_N_4_ formation has been extensively studied. Literature suggests that precursors such as urea and thiourea undergo stepwise condensation to form melamine, releasing ammonia and removing sulfur species in the process, while dicyandiamide directly condenses into melamine. With increasing temperature, the tri-s-triazine (heptazine) units formed from melamine undergo further polymerization to yield the extended g-C_3_N_4_ network [9,33,37]. This temperature-driven condensation not only determines the degree of polymerization but also dictates the resulting material’s textural characteristics and mechanical properties.

### 2.2. Solvothermal/Hydrothermal Method

The solvothermal and hydrothermal methods for synthesizing graphitic carbon nitride (g-C_3_N_4_) involve dissolving or dispersing nitrogen-rich precursors—such as melamine, urea, or dicyandiamide—in solvents such as water, acetonitrile, methanol, or ethanol. The resulting mixture is then subjected to high temperature and pressure within a sealed autoclave, where controlled reaction conditions promote the nucleation and growth of g-C_3_N_4_ crystals with tunable structures and physicochemical properties [38,39].

**Figure 6:**
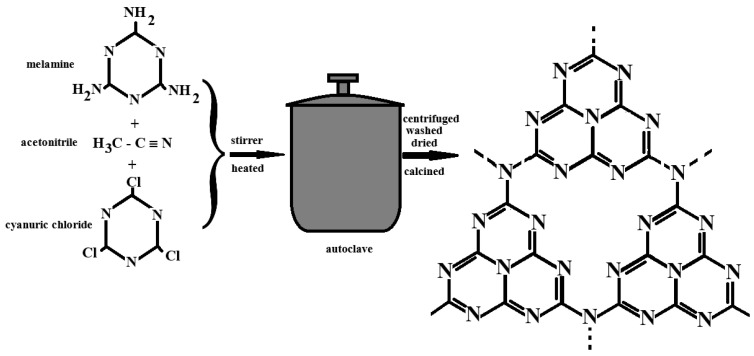
Schematic representation using the solvothermal/hydrothermal method to synthesize g-C_3_N_4_ [6,43,44].

These approaches offer significant advantages over conventional thermal polymerization, as they enable precise control over the particle size, morphology, and crystallinity of the final product, while also improving its purity and uniformity [40,41,42]. A schematic representation of the solvothermal synthesis process is provided in Figure 6.

Abdullahi et al. [6] synthesized g-C_3_N_4_ using acetonitrile as the solvent and examined the influence of reaction temperature (160 °C, 180 °C, and 200 °C) on material properties. Their results showed that synthesis at 200 °C enhanced both the optical absorption and photocatalytic efficiency of the product [6]. Similarly, Kunhikrishnan and co-workers [43] reported the hydrothermal synthesis of g-C_3_N_4_ using a mixture of melamine, methanol, and nitric acid. The homogeneous precursor solution was transferred into an autoclave and maintained at 80 °C for 12 h. After reaction, the obtained solid was centrifuged, washed with methanol, and further heat-treated at 100 °C for 6 h to improve structural rigidity. Characterization by X-ray diffraction (XRD) and scanning electron microscopy coupled with energy-dispersive X-ray spectroscopy (SEM-EDX) confirmed the successful formation of g-C_3_N_4_, displaying an acicular morphology with minor agglomerations and no detectable impurities, suitable for subsequent composite fabrication [43].

Other researchers synthesized spherical g-C_3_N_4_ particles using a mixture of dicyandiamide, cyanuric chloride, and acetonitrile. The solution was stirred for 24 h, transferred into an autoclave, and heated at 180 °C for another 24 h. The resulting material was washed with deionized water and dried under vacuum at 60 °C for 6 h, yielding uniform spherical structures [44]. Montigaud et al. [45] further reported a solvothermal reaction between melamine and hydrazine, which facilitated the formation of a graphite-like structure closely related to the C_3_N_4_ framework [45]. Beyond hydrothermal and solvothermal approaches, additional synthesis routes have been explored to tailor the morphology and performance of g-C_3_N_4_. These include high-energy microwave irradiation, sol–gel processing, hard- and soft-template methods, chemical vapor deposition (CVD), and electrochemical deposition. Each of these strategies offers distinct advantages in controlling crystallinity, porosity, and surface functionality, making g-C_3_N_4_ a highly adaptable material for diverse research and technological applications [39,40,46].

### 2.3. Morphology of g-C_3_N_4_

**Figure 7:**
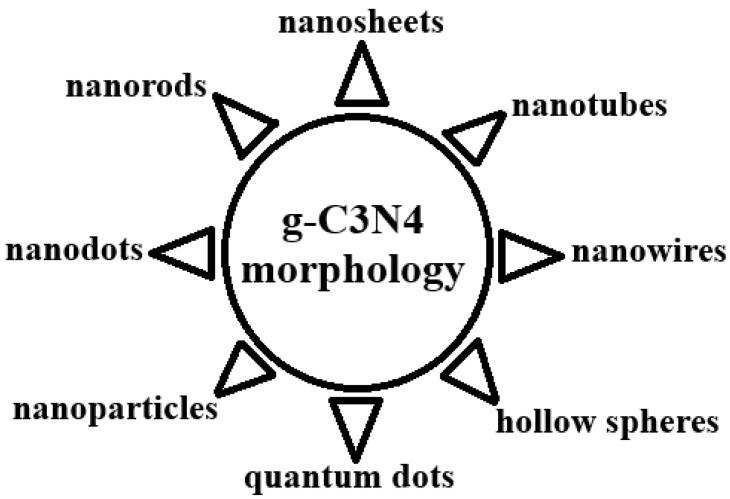
Potential application in nanotechnology [9,25,46,47].

Various morphologies reported in the specialized literature are illustrated in Figure 7. Shenthilkumar and colleagues, Wang et al. [46,47] emphasized in their research that g-C_3_N_4_ exhibits a wide range of nanoarchitectures, which can be classified by dimensionality into 0D (quantum dots, nanodots, hollow spheres), 1D (nanorods, nanowires), 2D (nanosheets, nanotubes), and 3D (mesoporous materials) structures. Similarly, Kyriakos et al. [9] confirmed that nanosheets correspond to 2D structures composed of chains of tri-s-triazine rings interconnected through hydrogen bonds.

The morphology of g-C_3_N_4_ plays a decisive role in determining key material properties, including photocatalytic performance, specific surface area, and electron transfer efficiency. These characteristics can be effectively tuned by controlling the particle shape and size through the appropriate selection of precursors and synthesis parameters. Wang et al. [48] illustrated, through representative schematic models, how varying synthesis conditions lead to distinct structural configurations of g-C_3_N_4_.

Further studies have shown that different synthesis strategies yield unique morphologies with specific advantages and targeted applications, such as photocatalysis, water splitting, environmental remediation, and sensor development [1,49]. This morphological versatility underscores the adaptability of g-C_3_N_4_ and its potential to meet diverse technological and environmental challenges.

## 3. g-C_3_N_4_ Activities

**Figure 8:**
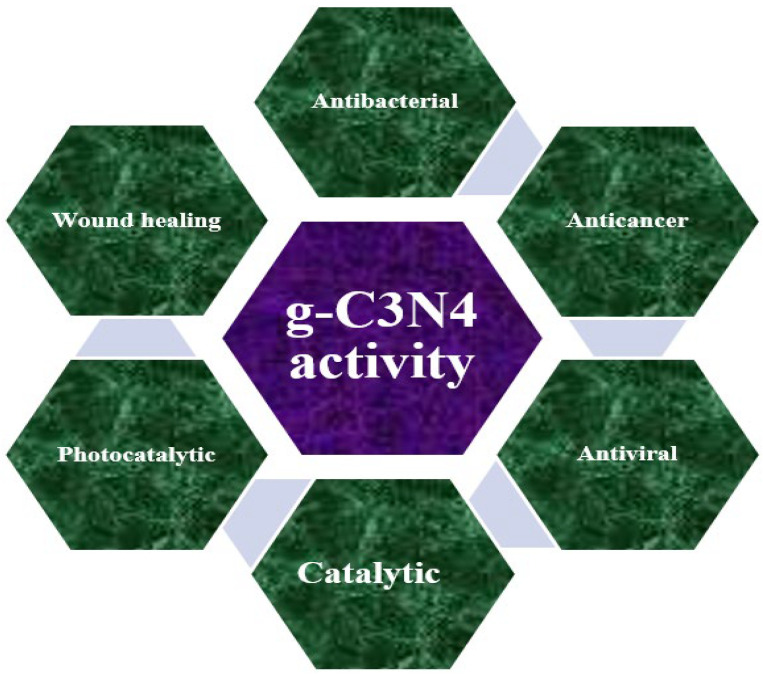
g-C_3_N_4_properties [40,50,51,52,53,54,55,56,57].

The oral cavity represents a fundamental component of the human body, acting as a critical interface between the external environment and internal physiological systems. Among its anatomical structures, the teeth play essential roles in mastication, facilitating proper digestion, enabling clear articulation during speech, and contributing to the aesthetics of a healthy smile. Given these functions, the equilibrium of the oral microbiota is highly dynamic, continuously influenced by physiological, environmental, and behavioral factors throughout an individual’s lifetime. Recent multidisciplinary investigations have revealed that graphitic carbon nitride (g-C_3_N_4_) possesses a diverse spectrum of functional properties—including antimicrobial, antiviral, catalytic, photocatalytic, and anticancer activities—as schematically illustrated in Figure 8. These characteristics open new prospects for translational research in oral health, a field in which applications of g-C_3_N_4_ remain relatively unexplored.

Liu et al. [50] synthesized g-C_3_N_4_ with photodynamic antibacterial properties targeting *Staphylococcus aureus* and *Streptococcus mutans* strains. Under visible light irradiation, the material generated reactive oxygen species (ROS), leading to the complete inactivation of both bacterial species within approximately 30 min [50]. Similarly, Thurston et al. [51] developed photoactive, urea-based g-C_3_N_4_ films exhibiting biocidal and sporicidal activity. When tested against *Escherichia coli*, *Staphylococcus aureus*, and *Bacillus anthracis* endospores, these films demonstrated strong antibacterial efficacy under 100 mJ of visible light exposure, although the *Bacillus anthracis* endospores exhibited greater resistance [51]. As well, regarding wound healing, g-C_3_N_4_based materials demonstrate the efficiency in killing bacteria by generating reactive oxygen species [40].

In terms of anticancer potential, Alonso et al. [52] evaluated g-C_3_N_4_ against glioblastoma cell lines (LN229 and SNB19) and noncancerous mouse embryonic fibroblasts. Their findings revealed substantial growth inhibition—approximately 85% in LN229 and 46.5% in SNB19 cells—while cytotoxic effects on normal cells remained minimal [52]. Likewise, Yoshira et al. [53] examined the antitumor efficacy of g-C_3_N_4_ in DU-145 (prostate cancer) and U87 (glioblastoma) cell lines, reporting significant decreases in cell viability within 48 h. The selectivity assays conducted on non-tumorigenic RAW 264.7 and HFF-1 cell lines confirmed that g-C_3_N_4_ displayed low toxicity toward healthy cells.

The antiviral activity of g-C_3_N_4_ under visible light irradiation was demonstrated by Zhang et al. [54], who emphasized its potential role in water disinfection. Their results indicated complete inactivation of waterborne viruses at an initial concentration of 8 log PFU/mL after 240 min of visible light exposure, with no subsequent viral regrowth. Similarly, Li et al. [55] confirmed that g-C_3_N_4_ exhibited durable and effective virucidal activity against the MS2 virus, reinforcing its promise as a viable material for antiviral and water purification applications [55].

It is well established that g-C_3_N_4_ possesses exceptional photocatalytic and catalytic capabilities, participating effectively in redox and activation reactions. Its photocatalytic performance derives from a relatively narrow band gap, which allows for efficient absorption of visible light [40,56]. Zhang et al. [57] synthesized porous g-C_3_N_4_ via a bubble-template pyrolysis technique employing urea and dicyandiamide as precursors. By increasing both the urea-to-dicyandiamide mass ratio and the calcination temperature, they enhanced the specific surface area from 5.4 to 60 m^2^/g. Consequently, the material demonstrated improved photocatalytic efficiency under visible light irradiation, showing high potential for the degradation of organic pollutants [57].

## 4. g-C_3_N_4_ in Oral Dentistry

Despite the growing interest in g-C_3_N_4_ across diverse biomedical and catalytic applications, studies exploring its integration into dental materials remain limited. These are still in the initial stage of development [58]. Recent innovations in dental composites have focused on incorporating photodynamic therapy (PDT) functionality to prevent bacterial colonization and improve oral health outcomes. In this context, g-C_3_N_4_ has emerged as a promising additive due to its photocatalytic and antimicrobial properties.

One notable approach involved embedding g-C_3_N_4_ into a BisGMA/TEGDMA dental resin matrix, resulting in a composite capable of achieving a bacterial elimination rate exceeding 99% against *Staphylococcus aureus* and *Streptococcus mutans*. The biocompatibility of the resin composites was assessed using an in vitro L929 fibroblast culture model. Cells were seeded onto the composite discs and incubated for 1–5 days, followed by live/dead staining to evaluate viability under LSCM, where green and red fluorescence indicated live and dead cells, respectively. Prolonged culture demonstrated sustained cell proliferation, indicating that the g-C_3_N_4_-based composites exhibit good biocompatibility and are therefore suitable for further antibacterial evaluation [50]. Importantly, the inclusion of g-C_3_N_4_ did not compromise the composite’s mechanical performance or biocompatibility, indicating its suitability for clinical application in restorative dentistry [50]. This study demonstrates the potential of g-C_3_N_4_-based composites to provide active antimicrobial protection while maintaining the functional requirements of dental restorative materials.

Beyond conventional resin composites, heterostructured materials incorporating g-C_3_N_4_ have also shown promise. For example, g-C_3_N_4−x_/Bi_2_O_3−y_ heterostructures exhibit significant photocatalytic and piezo-photocatalytic activity, suggesting potential applications in tooth whitening and antibacterial treatments. These materials leverage synergistic mechanisms to generate reactive oxygen species under light or mechanical stimulation, enhancing their therapeutic efficacy [59].

Furthermore, Jiawen and Xing [60] developed a novel CeO_2_–g-C_3_N_4_–BA nanoparticles (NPs) nanocomposite, which demonstrated strong antibacterial activity while simultaneously promoting alveolar bone regeneration in periodontitis animal models. The composite enhanced osteoblast proliferation and differentiation and improved cellular antioxidant capacity, highlighting the dual functionality of g-C_3_N_4_-based nanomaterials in both antimicrobial defense and tissue regeneration [60]. The promotion of periodontal bone tissue repair was assessed using in vitro co-culture models and animal models of periodontitis, employing ROS detection methods and analyses of cellular signaling pathways. The authors demonstrated that the nanocomposite modulates the Nrf2/HO-1 pathway, enhances cellular antioxidant capacity, stimulates osteoblast activity, and supports alveolar bone repair and regeneration while exhibiting excellent biocompatibility. In addition, it mitigates inflammatory responses [60]. Collectively, these findings provide a theoretical basis for the nanocomposite’s potential application in the treatment of periodontitis. Collectively, these studies illustrate the versatility of g-C_3_N_4_ as a multifunctional additive in dental materials. By combining antimicrobial, photocatalytic, and regenerative properties, g-C_3_N_4_-based composites represent a promising avenue for next-generation restorative and therapeutic dental applications. Future research should focus on optimizing these materials for clinical use, exploring long-term stability, controlled release mechanisms, and synergistic effects with other bioactive components to enhance oral health outcomes.

## 5. Conclusions and Perspectives

This review provided a comprehensive overview of graphitic carbon nitride (g-C_3_N_4_), including its fundamental structural components, common synthesis methods, and applications across diverse scientific and technological fields. Atomic-level investigations using AFM and STM revealed detailed structural characteristics of Highly Oriented Pyrolytic Graphite (HOPG), Graphene Oxide, and graphite nitride, offering valuable insights into their morphology and surface properties.

Despite its broad applicability, a notable gap exists in research exploring the use of g-C_3_N_4_ within the dental field. Given its unique combination of physicochemical properties, photocatalytic activity, and antimicrobial potential, g-C_3_N_4_ represents a promising candidate for the development of novel dental materials. Future studies should focus on leveraging these properties to design advanced composites for oral healthcare applications, including the prevention and treatment of oral tissue disorders. The current scarcity of publications in this area highlights an important opportunity to bridge fundamental material research with translational applications in dentistry, particularly in the context of antibacterial therapies and photodynamic treatment strategies.

In conclusion, g-C_3_N_4_ holds substantial promise as a multifunctional material in oral healthcare, bridging its intrinsic physicochemical advantages with therapeutic potential. Addressing the current research gaps could lead to innovative solutions for infection control, tissue regeneration, and photodynamic applications in dentistry, positioning g-C_3_N_4_ as a cornerstone of future oral biomaterials research.

## Figures and Tables

**Table 1:** g-C_3_N_4_ parameters and product structure [27,28,29,30,31,32,33,34,35,36].

Precursors	Temperature (°C)	Heating Time (h)	Ramp Rate (°C/min)	Organoleptic Properties	Surface Area	Structural Type	References
Melamine	550 °C 700 °C	4	5 °C/min	yellow powder	38.38 m^2^/g at 700 °C 10.38 m^2^/g at 500 °C	bulk g-C_3_N_4_	[27,28,29,30]
Dicyanamide	500 °C 550 °C	4 2	10 °C/min 15 °C/min	yellow material	9.82 m^2^/g 14.87 m^2^/g	porous structure, more amino functional group smore stable chemical bond structures	[30,31,32]
Urea	550 °C	2, 3, 4, 5	5 °C/min	light-yellow product	108.83 m^2^/g	layered nanostructure, laminar morphology -orm stacked CN sheets in a complex reaction enhanced crystallinity	[9,27,31,33,34,35]
Thiourea	580 °C	4	2.3 °C/min	Pale yellow	92.8 m^2^/g larger specific surface area	nanosheet structures faster photoinduced electron–hole pair transfer efficiency	[36]

## Data Availability

The original contributions presented in the study are included in the article; further inquiries can be directed to the corresponding author.

## Abbreviations

The following abbreviations are used in this manuscript:

AFM	Atomic Force Microscopy
STM	Scanning Tunneling Microscopy
HOPG	Highly Oriented Pyrolytic Graphite
g-C_3_N_4_	Graphitic carbon nitride
